# Fano feature induced by a bound state in the continuum via resonant state expansion

**DOI:** 10.1038/s41598-020-70654-2

**Published:** 2020-08-13

**Authors:** Pavel S. Pankin, Dmitrii N. Maksimov, Kuo-Ping Chen, Ivan V. Timofeev

**Affiliations:** 1 Kirensky Institute of Physics, Federal Research Center KSC SB RAS, Krasnoyarsk, Russia 660036; 2 Siberian Federal University, Krasnoyarsk, Russia 660041; 3 Institute of Imaging and Biomedical Photonics, National Chiao Tung University, Tainan, 71150 Taiwan, ROC

**Keywords:** Photonic crystals, Nanophotonics and plasmonics

## Abstract

We consider light scattering by an anisotropic defect layer embedded into anisotropic photonic crystal in the spectral vicinity of an optical bound state in the continuum (BIC). Using a resonant state expansion method we derive an analytic solution for reflection and transmission amplitudes. The analytic solution is constructed via a perturbative approach with the BIC as the zeroth order approximation. The solution is found to describe the collapsing Fano feature in the spectral vicinity of the BIC. The findings are confirmed via comparison against direct numerical simulations with the Berreman transfer matrix method.

## Introduction

Theoretical insight into resonant response from optical systems, including photonic-crystalline resonators^1^ and resonant metasurfaces^2^, is of big importance in photonics^3,4^. Very unfortunately only a few systems generally allow for a tractable analytic solution providing intuitively clear and mathematically exact picture, such as, e.g., the celebrated Mie–Lorenz theory^5^. Thus, in the field of optics the resonant scattering quite often can only be understood in terms of the temporal coupled mode theory (TCMT)^6–8^. The TCMT is a phenomenological approach that maps the scattering problem onto a system of field driven lossy oscillators. Mathematically, the problem is cast in the form of a system of linear differential equations. The coefficients of the system account for both “internal” modes of the resonant structure as well as for the coupling of the “internal” modes to incoming and outgoing waves. The interaction with the impinging light is understood in terms of “coupled modes” which are populated when the system is illuminated from the far-zone. The elegance of the TCMT is in its simplicity and the relative ease in establishing important relationships between the phenomenological coefficients solely from the system’s symmetries and conservation laws^7–10^. However, despite its numerous and successful applications, the TCMT generally relies on a set of fitting parameters. Moreover, the mathematical foundations of the TCMT remain vague since the theory neither gives an exact definition of the “coupled mode”, nor a clear recipe for such a “coupled mode” to be computed numerically.

Historically, the problem of coupling between the system’s eigenmodes to the scattering channels with the continuous spectrum has attracted a big deal of attention in the field of quantum mechanics^11–13^. One of the central ideas was the use of the Feshbach projection method^12,14^ for mapping the problem onto the Hilbert space spanned by the eigenstates of the scattering domain isolated from the environment. Such approaches have met with a limited success in application to various wave related set-ups, including quantum billiards^15–17^, tight-binding models^18^, potential scattering^19^, acoustic resonators^20^, nanowire hetrostructures^21^ and, quite recently, dielectric resonators^22^. Besides its mathematical complexity there are two major problems with the Feshbach projection method: First, the eigenmodes of the isolated systems are in general not known analytically; therefore, some numerical solver has most often to be applied. Furthermore, the computations of such eigenmodes requires some sort of artificial boundary condition on the interface between the scattering domain and the outer space. Quite remarkably the convergence of the method is shown to be strongly affected by the choice of the boundary condition on the interface^16,23,24^.

In the recent decades we have witnessed the rise of efficient numerical solvers utilizing perfectly matched layer (PML) absorbing boundary conditions^25,26^. The application of perfectly matched layer has rendered numerical modelling of wave propagation in open optical, quantum, and acoustic systems noticeably less difficult allowing for direct full-wave simulations even in three spatial dimensions. On the other hand, the application of PML also made it possible to compute the eigenmodes and eigenfrequencies of wave equations with refletionless boundary conditions. Such eigenmodes come under many names including quasinormal modes^4^, Gamow states^27^, decaying states^28^, leaky modes^29^, and resonant states^30^. The availability of the resonant states has naturally invited applications to solving Maxwell’s equations via series expansions giving rise to a variety of resonant state expansion (RSE) methods^4^. One problem with the resonant states is that they are not orthogonal in the usual sense of convolution between two mode shapes with integration over the whole space^13,31^. This can be seen as a consequence of exponential divergence with the distance from the scattering center^4^. Fortunately, both of the normalization and orthogonality issues have recently been by large resolved with different approaches, most notably through the PML^32^, and the flux-volume (FV)^28,30^ normalization conditions.

In this paper we propose a RSE approach to the problem of light scattering by an anisotropic defect layer (ADL) embedded into anisotropic photonic crystal (PhC) in the spectral vicinity of an optical bound state in the continuum (BICs). The optical BICs are peculiar localized eigenmodes of Maxwell’s equations embedded into the continuous spectrum of the scattering states^33,34^. One remarkable property of the BICs is the emergence of a collapsing Fano features induced by the high-quality resonant states in the spectral vicinity of the BIC proper^35–40^. The sensitivity of the optical response to the parameters of the incident light allows for a fine control of Fano line-shapes making the optical BICs an efficient instrument in design of narrowband optical filters^41–44^. Although BICs are ubiquitous^33,34^ in various optical systems, the system under scrutiny is the only one allowing for an exact full-wave analytic solution for an optical BIC^45^. By matching the general solution of Maxwell’s equation within the ADL to both evanescent and propagating solutions in the PhC^46–49^ we find the eigenfield and eigenfrequency of the resonant mode family limiting to the BIC under variation of a control parameter. Next, for finding the scattering spectra we apply the spectral representation of Green’s function in terms the FV-normalized resonant states^30^. This is a well developed approach which has already been applied to both two^50^- and three^51^-dimensional optical systems. The approach has also been recently extended to magnetic, chiral, and bi-anisotropic optical materials^52^ as well as potential scattering in quantum mechanics^53^. Remarkably, so far RSE methods have been mostly seen as a numerical tool. Here we show how a perturbative analytic solution can be constructed in a closed form within the RSE framework. Such a perturbative solution uses the BIC as the zeroth order approximation and, very importantly, is capable of describing the collapsing Fano resonance^35–40^ in the spectral vicinity of the BIC. We shall see that the analytic solution matches the exact numerical result to a good accuracy.

## The system

**Figure 1:**
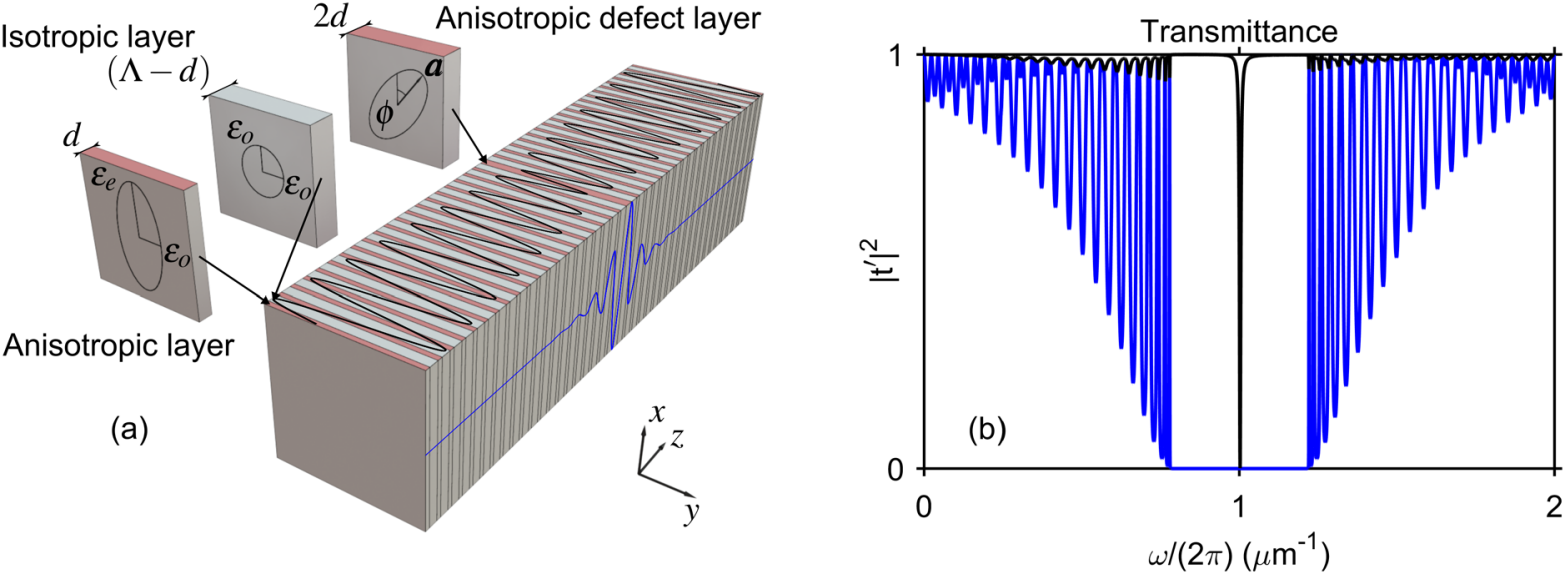
(**a**) One-dimensional PhC structure stacked of alternating layers of an isotropic dielectric material with permittivity $\epsilon _o$ (gray) and an anisotropic material with the permittivity components $\epsilon _o$ and $\epsilon _e$ (pink). An anisotropic defect layer with a tuneable permittivity tensor is inserted in the center of the structure. The analytic solution for the quasi-BIC mode profile is plotted on top and right sides of the stack: the *x*-wave component Re$(E_x)$—blue, the *y*-wave component Re$(E_y)$—black. (**b**) The transmittance spectra $|t^{\prime }|^2$ of *x*- (blue) and *y*-waves (black) for PhC structure from (**a**) calculated with Berreman’s method. The parameters are $\epsilon _e = 4$, $\epsilon _o = 1$, $d = 0.125 \ \upmu \text {m}$, $(\Lambda - d) = 0.250 \ \upmu \text {m}$, tilt angle $\phi = \pi /9$ (**a**), $\phi = \pi /18$ (**b**).

The system under scrutiny is composed of an ADL with two anisotropic PhC arms attached to its sides as shown in Fig. 1a. Each PhC arm is a one-dimensional PhC with alternating layers of isotropic and anisotropic dielectric materials. The layers are stacked along the *z*-axis with period $\Lambda$. The isotropic layers are made of a dielectric material with permittivity $\epsilon _o$ and thickness $\Lambda -d$. The thickness of each anisotropic layer is *d*. The anisotropic layers have their principal dielectric axes aligned with the *x*, *y*-axes with the corresponding permittivity component principal dielectric constants $\epsilon _{e}$, $\epsilon _{o}$, but the principal axes of the ADL are tilted with respect of the principal axes of the PhC arms as shown in Fig. 1a. Propagation of the monochromatic electromagnetic waves is controlled by Maxwell’s equations of the following form^45^

1 $$\begin{aligned} \left\{ \begin{array}{cc} 0 &{} \quad \nabla \times \\ -\nabla \times &{} \quad 0 \end{array}\right\} \left\{ \begin{array}{c} {\varvec{E}} \\ {\varvec{H}} \end{array} \right\} = -ik_0 \left\{ \begin{array}{c} {\hat{\epsilon }} {\varvec{E}} \\ {\varvec{H}} \end{array}\right\} , \end{aligned}$$

where $\varvec{E}$ is the electric vector, $\varvec{H}$ is the magnetic vector, $k_0 = \omega /c$ is the wave number in vacuum with *c* as the speed of light, and, finally, ${\hat{\epsilon }}$ is the dielectric tensor. The orientation of the ADL optical axis is determined by the unit vector

2 $$\begin{aligned} \varvec{a} = \{\cos {(\phi )}, \sin {(\phi )}, 0\}^{\dagger }, \end{aligned}$$

as shown in Fig. 1a. Since the reference frame is aligned with the optical axes in the PhC, the dielectric tensor is diagonal everywhere out of the ADL. Given that $\varvec{a}$ is specified by the tilt angle $\phi$, in the ADL it takes the following form

3 $$\begin{aligned} {\hat{\epsilon }} = \left\{ \begin{array}{cc} \epsilon _e \cos ^2 (\phi ) + \epsilon _o \sin ^2 (\phi ) &{} \quad \sin {(2\phi )} \; (\epsilon _e-\epsilon _o)/2 \\ \sin {(2\phi )} \; (\epsilon _e-\epsilon _o)/2 &{} \quad \epsilon _e \sin ^2 (\phi ) + \epsilon _o \cos ^2 (\phi ) \end{array} \right\} . \end{aligned}$$

In this paper we restrict ourselves with the normal incidence, i.e. the Bloch wave vector is aligned with the *z*-axis in Fig. 1a. The dispersion of waves in the PhC arms depends on polarization. For the *x*-polarized waves (*x*-waves) the dispersion relationship is that of a one-dimensional PhC^46–49^

4 $$\begin{aligned} \cos {(K \Lambda )} = \cos {(k_{e} d)} \cos {[k_{o} (\Lambda - d)]} - \frac{1 + r_{o e}^2}{1 - r_{o e}^2} \sin {(k_{e} d)} \sin {[k_{o} (\Lambda - d)]}, \end{aligned}$$

where *K* is the Bloch wave number,

5 $$\begin{aligned} k_{e} = k_0 \sqrt{\epsilon _e} = k_0 n_e, \ k_{o} = k_0 \sqrt{\epsilon _o} = k_0 n_o, \end{aligned}$$

and the Fresnel coefficient $r_{o e}$ is given by

6 $$\begin{aligned} r_{o e} = \frac{k_{o} - k_{e}}{k_{o} + k_{e}}. \end{aligned}$$

Equation (4) defines the band structure for the *x*-waves with the condition $|\cos {(K \Lambda )}| = 1$ corresponding to the edges of the photonic band gap in which the wave propagation is forbidden. In Fig. 1a we demonstrate the transmittance spectrum of the system with 20 bi-layers in each PhC arms; the overall system being submersed in air. One can see that for the *x*-waves the transmittance is zero within the band gap. On the other hand the PhC arms are always transparent to the *y*-polarized waves (*y*-waves) with dispersion $k_o=\epsilon _o k_0$. Notice, though, that the *y*-waves transmittance exhibits a sharp dip at the center of the band gap. This dip is due to a high quality resonant mode predicted in^45^. Although the line shape is symmetric, the dip, nonetheless, can be attributed to a Fano resonance as the transmittance reaches zero at the center of the band gap indicating a full destructive interference between two transmission paths. In this paper we set a goal of finding the analytic solution describing the Fano anomaly in the band gap.

## Resonant eigenmode and bound state in the continuum

The resonant states are the eigenmodes of Maxwell’s equations (1) with reflectionless boundary conditions in the PhC arms. The equation for resonant eigenfrequencies can be obtained by matching the general solution in the ADL to the outgoing, both propagating and evanescent, waves in the PhC arms. Let us start from the general solution in the ADL.

### General solution in the ADL

First, the unit vector along the propagation direction is defined as

7 $$\begin{aligned} \varvec{\kappa }^{\scriptscriptstyle (\pm )} = [0, 0, \pm 1], \end{aligned}$$

where the symbol ± is used to discriminate between forward and backward propagating waves with respect to the orientation of the *z*-axis. The ADL supports two types of electromagnetic waves of different polarization. The *e*-waves with wavenumber $k_e=\epsilon _e k_0$ are polarized along the director $\varvec{a}$, Eq. (2), while the *o*-waves with wavenumber $k_o=\epsilon _o k_0$ have their electric vector perpendicular to both $\varvec{a}$ and $\varvec{\kappa }$, as seen from Fig. 1. The electric and magnetic vectors of the *e*-wave can be written as

8 $$\begin{aligned} \varvec{E}_{e}^{\scriptscriptstyle (\pm )} = E_{e}^{\scriptscriptstyle (\pm )} \varvec{a}, \ \ \varvec{H}_{e}^{\scriptscriptstyle (\pm )} = \frac{k_e}{k_0}\left[ \varvec{\kappa }^{\scriptscriptstyle (\pm )} \times \varvec{E}_{e}^{\scriptscriptstyle (\pm )} \right] , \end{aligned}$$

where $E_{e}^{\scriptscriptstyle (\pm )}$ are unknown amplitudes. At the same time for *o*-waves we have

9 $$\begin{aligned} \varvec{E}_{o}^{\scriptscriptstyle (\pm )} = E_{o}^{\scriptscriptstyle (\pm )} \left[ \varvec{a} \times \varvec{\kappa }^{\scriptscriptstyle (\pm )} \right] , \ \ \varvec{H}_{o}^{\scriptscriptstyle (\pm )} = \frac{k_o}{k_0}\left[ \varvec{\kappa }^{\scriptscriptstyle (\pm )} \times \varvec{E}_{o}^{\scriptscriptstyle (\pm )} \right] , \end{aligned}$$

where $E_{o}^{\scriptscriptstyle (\pm )}$ are again unknown amplitudes. The general solution of equations (1) in the ADL, $\ z \in [-d,\ d]$, is written as a sum of the forward and backward propagating waves

10 $$\begin{aligned} \varvec{E} = \sum _{j=o,e} \left( \varvec{E}_{j}^{\scriptscriptstyle (+)} e^{i k_{j} z} + \varvec{E}_{j}^{\scriptscriptstyle (-)} e^{-i k_{j} z} \right) ,\ \ \varvec{H} = \sum _{j=o,e} \left( \varvec{H}_{j}^{\scriptscriptstyle (+)} e^{i k_{j} z} + \varvec{H}_{j}^{\scriptscriptstyle (-)} e^{-i k_{j} z} \right) . \end{aligned}$$

### General solution in the PhC arms

The general solution of Maxwell’s equations (1) in the PhC arms is also written as a sum of forward and backward propagating waves. For the *x*-waves the field components $E_x$ and $H_y$ in the isotropic layer with the cell number *m*, $\ z \in [d + m \Lambda ,\ (m + 1) \Lambda ]$, are written as

11 $$\begin{aligned} \begin{aligned}E_{x}^{(m)} &= e^{iK \Lambda m}\left[ A^{\scriptscriptstyle (+)}_x e^{i k_{o} (z - d - m \Lambda )} + A^{\scriptscriptstyle (-)}_x e^{-i k_{o} (z - d - m \Lambda )}\right] , \\H_{y}^{(m)} &= \frac{k_{o}}{k_0} e^{iK \Lambda m}\left[ A^{\scriptscriptstyle (+)}_x e^{i k_{o} (z - d - m \Lambda )} - A^{\scriptscriptstyle (-)}_x e^{-i k_{o} (z - d - m \Lambda )}\right] . \end{aligned} \end{aligned}$$

In the anisotropic layer with the cell number *m*, $\ z \in [(m+1) \Lambda ,\ d + (m + 1) \Lambda ]$, we have

12 $$\begin{aligned} \begin{aligned}E_{x}^{(m)} &= e^{iK \Lambda m}\left[ B^{\scriptscriptstyle (+)}_x e^{i k_{e} (z - m\Lambda -\Lambda )} + B^{\scriptscriptstyle (-)}_x e^{-i k_{e} (z - m\Lambda -\Lambda )}\right] , \\H_{y}^{(m)} &= \frac{k_{e}}{k_0} e^{iK \Lambda m}\left[ B_x^{\scriptscriptstyle (+)} e^{i k_{e} (z - m\Lambda -\Lambda )} - B_x^{\scriptscriptstyle (-)} e^{-i k_{e} (z - m\Lambda -\Lambda )}\right] . \end{aligned} \end{aligned}$$

By applying the continuity condition for the tangential field components the solutions (11) and (12) are matched on the boundary between the anisotropic layer in the $(m-1)_{\mathrm{th}}$ cell and the isotropic layer in $m_{\mathrm{th}}$ cell, $\ z = d + m \Lambda$, as well as on the boundary between the layers in the $m_{\mathrm{th}}$ cell, $\ z = (m + 1)\Lambda$. This gives us a system of four equations for four unknowns, $A^{\scriptscriptstyle (+)}, A^{\scriptscriptstyle (-)}, B^{\scriptscriptstyle (+)}, B^{\scriptscriptstyle (-)}$. After solving for $B^{\scriptscriptstyle (+)}$ and $B^{\scriptscriptstyle (-)}$, this system can be reduced to the following two equations

13 $$\begin{aligned} \left\{ \begin{aligned} A^{\scriptscriptstyle (+)} \left[ e^{i k_{o} (\Lambda - d)} - e^{iK \Lambda } e^{-i k_{e} d}\right] - A^{\scriptscriptstyle (-)} r_{o e}\left[ e^{-i k_{o} (\Lambda - d)} - e^{iK \Lambda } e^{-i k_{e} d}\right] = 0, \\ -A^{\scriptscriptstyle (+)} r_{o e} \left[ e^{i k_{o} (\Lambda - d)} - e^{iK \Lambda } e^{i k_{e} d}\right] + A^{\scriptscriptstyle (-)} \left[ e^{-i k_{o} (\Lambda - d)} - e^{iK \Lambda } e^{i k_{e} d}\right] = 0, \end{aligned}\right. \end{aligned}$$

where $r_{oe}$ is given by Eq. (6). One can easily check that Eq. (13) is only solvable when *K* satisfies the dispersion relationship (4).

In contrast to the *x*-waves, for the outgoing *y*-waves in the right PhC arms the solution is simple

14 $$\begin{aligned} \begin{aligned}E_{y} &= -C^{\scriptscriptstyle (+)} e^{i k_{o} (z - d)}, \\H_{x} &= \frac{k_{o}}{k_0} C^{\scriptscriptstyle (+)} e^{i k_{o} (z - d)}. \end{aligned} \end{aligned}$$

Notice that so far we have not written down the solution in the left PhC arm. The direct application of that solution can be avoided by using the mirror symmetry of the system. Here, in accordance with ref^45^ we restrict ourselves with the antisymmetric case

15 $$\begin{aligned} \varvec{E}(z) = - \varvec{E}(-z). \end{aligned}$$

The generalization onto the symmetric case is straightforward.

### Wave matching

Now we have all ingredients for finding the field profile of the antisymmetric resonant eigenmodes. By matching equation (10) to both Eqs. (11) and (14) on the interface between the ADL and the right PhC arm and using Eqs. (13, 15) one obtains eight equations for eight unknown variables $E_{e}^{\scriptscriptstyle (+)}, E_{e}^{\scriptscriptstyle (-)}, E_{o}^{\scriptscriptstyle (+)}, E_{o}^{\scriptscriptstyle (-)}, A^{\scriptscriptstyle (+)}, A^{\scriptscriptstyle (-)}, C^{\scriptscriptstyle (+)}, K$. After some lengthy and tedious calculations one finds that the system is solvable under the following condition

16 $$\begin{aligned} \frac{\xi e^{i k_{o} (\Lambda - d)} - r_{o e} e^{-i k_{o} (\Lambda - d)}}{\xi e^{-i k_{e} d} - r_{o e} e^{-i k_{e} d}} - \frac{e^{-i k_{o} (\Lambda - d)} - \xi r_{o e} e^{i k_{o} (\Lambda - d)}}{e^{i k_{e} d} - \xi r_{o e} e^{i k_{e} d}} = 0, \end{aligned}$$

where

17 $$\begin{aligned} \xi = -e^{2i k_{o} d}\sin ^2{(\phi )} + \frac{r_{oe} - e^{2i k_{e} d}}{1 - r_{oe} e^{2i k_{e} d}} \cos ^2{(\phi )}. \end{aligned}$$

Taking into account Eq. (5) we see that the above formulae represent the transcendental equation for complex eigenvalues, $k_0$ of the Maxwell’s equations (1). The analytic solution for the electromagnetic field within the ADL can be evaluated through the following formulae

18 $$\begin{aligned} \begin{aligned}E_{o}^{\scriptscriptstyle (+)} &= E_{o}^{\scriptscriptstyle (-)}, \ E_{e}^{\scriptscriptstyle (-)} = \zeta E_{o}^{\scriptscriptstyle (-)}, \ E_{o}^{\scriptscriptstyle (+)} = -\zeta E_{o}^{\scriptscriptstyle (-)}, \ E_{o}^{\scriptscriptstyle (-)} = \frac{i A}{2 n_e \zeta },\\\zeta &= - \frac{e^{-i k_{o} d} t_{oe} \cos {(\phi )}}{(e^{-i k_{e} d} - r_{oe} e^{i k_{e} d}) \sin {(\phi )}}, \ t_{oe} = \frac{2k_{o}}{k_{o} + k_{e}}. \end{aligned} \end{aligned}$$

Substituting (18) into Eq. (10) one finds the profile of the resonant eigenmode within the ADL

19 $$\begin{aligned} \begin{aligned}E_x &= \frac{A}{n_e} \sin {(k_e z)} \cos {(\phi )} - \frac{A}{n_e \zeta } \sin {(k_o z)} \sin {(\phi )}, \\E_y &= \frac{A}{n_e} \sin {(k_e z)} \sin {(\phi )} + \frac{A}{n_e \zeta } \sin {(k_o z)} \cos {(\phi )}. \end{aligned} \end{aligned}$$

The amplitude *A* has to be defined from a proper normalization condition. We mention that in limiting case $\phi \rightarrow 0$ the $\zeta \rightarrow \infty$ and fields $E_x = (A/n_e) \sin {(k_e z)}$, and $E_y = 0$ coincide with exact solution for BIC (8), (21) from our previous work^45^. The obtained eigenfield are plotted in Fig. 1a. One can see that though the *y*-component is localized due to the band gap, the *x*-component grows with the distance from the ADL as it is typical for resonant eigenstates^4^.

### Perturbative solution

Equations (16, 17) are generally not solvable analytically. There is, however, a single tractable perturbative solution in the case of quarter-wave optical thicknesses of the layers

20 $$\begin{aligned} k_{o} (\Lambda - d) = k_{e} d = \frac{k_0 \lambda _{\scriptscriptstyle PBG}}{4} = \frac{\pi \omega }{2 \omega _{\scriptscriptstyle PBG}}, \end{aligned}$$

where $\omega _{\scriptscriptstyle PBG}$ is the center frequency of photonic band gap, and $\lambda _{\scriptscriptstyle PBG}$ is the corresponding wavelength. In our previous work^45^ we found an exact solution for $\phi =0$. Here by applying a Taylor expansion of equations (16, 17) in powers of the tilt angle $\phi$ we found approximate solutions for both resonant eigenfrequency and resonant eigenomode. By writing the resonant eigenfrequency as

21 $$\begin{aligned} \omega _r={\tilde{\omega }}-i \gamma , \end{aligned}$$

where both ${\tilde{\omega }}$ and $\gamma$ are real and positive, and substituting into Eqs. (16, 17) one finds

22 $$\begin{aligned} \begin{aligned}{\tilde{\omega }} &= \omega _{\scriptscriptstyle PBG} + \frac{\omega _{\scriptscriptstyle PBG}}{\pi } q (1 - q) \sin {(\pi q)} \cdot \phi ^{2} + {\mathscr{O}}(\phi ^4)\\\gamma &= \frac{2 \omega _{\scriptscriptstyle PBG}}{\pi } q (1 - q) \cos ^2{(\pi q/2)} \cdot \phi ^{2}+ {\mathscr{O}}(\phi ^4). \end{aligned} \end{aligned}$$

where $q = n_{o}/n_{e}$. Notice that the imaginary part of $\omega$ vanishes if $\phi =0$. Thus, the system supports an antisymmetric BIC with the frequency $\omega _{\scriptscriptstyle BIC}=\omega _{\scriptscriptstyle PBG}$. That BIC was first reported in our previous work^45^. We address the reader to the above reference for detailed analysis of the BIC and the plots visualizing its eigenfields. For the further convenience we also introduce the resonant vacuum wave number as

23 $$\begin{aligned} k_r = (\omega _r - i \gamma )/c = k_{\scriptscriptstyle BIC}\left[ 1 + \alpha \cdot \phi ^2+ {\mathscr{O}}(\phi ^4)\right] , \end{aligned}$$

where the complex valued $\alpha$ is implicitly defined by Eq. (22) and $k_{\scriptscriptstyle BIC}=\omega _{\scriptscriptstyle BIC}/c$. Finally, expanding (19) into the Taylor series in $\phi$ we find the following expression for the resonant eigenmode profile within the ADL

24 $$\begin{aligned} \varvec{E}_r(z) = \frac{A}{n_e} \left\{ \begin{array} {c} \sin {\left( \frac{\pi z}{2 d}\right) } + {\mathscr{O}}(\phi ^2) \\ \left[ \sin {\left( \frac{\pi z}{2 d}\right) } + ie^{\frac{i\pi q}{2}} \sin {\left( \frac{\pi q z}{2 d}\right) } \right] \cdot \phi + {\mathscr{O}}(\phi ^3) \end{array}\right\} . \end{aligned}$$

Notice that ${\varvec{E}}_r$ can be handled as $2\times 1$ vector since $E_z=0$.

### Normalization condition

There are several equivalent formulations of the FV normalization condition^30,50,52^. Here we follow^50^, writing down the FV normalization condition through analytic continuation ${\varvec{E}}(z,k)$ of the resonant eigenmode ${\varvec{E}}_r(z)$ around the point $k=k_r$

25 $$\begin{aligned} \int \limits _{-d}^{d}{\varvec{E}}_r\cdot {\hat{\epsilon }}{\varvec{E}}_{r}dz - \lim _{k\rightarrow k_r}\left\{ \frac{2\left[ {\varvec{E}}_r(d)\cdot \partial _z{\varvec{E}}(d,k)- {\varvec{E}}(d,k)\cdot \partial _z{\varvec{E}}_{r}(d) \right] }{k_{r}^2-k^2}\right\} =1. \end{aligned}$$

At the first glance the “flux” term in Eq. (25) differs from that in^50^ by the factor of 2; this is because the “flux” term is doubled to account for both interfaces $z=\pm d$. For the resonant eigenmode $\varvec{E}_r(z)$ found in the previous subsection the amplitude *A* corresponding equation (25) can be found analytically we the use of Eq. (24). This would, however, result in a very complicated expression. Fortunately, we shall see later on that in our case we do not need the general expression for *A* in the second order perturabtive solution consistent with Eq. (22). By a close examination of Eq. (24) one can see that the the Taylor expansion of *A* can only contain even powers of $\phi$. Thus, for the further convenience we can write

26 $$\begin{aligned} A = \frac{1}{\sqrt{F+B\cdot \phi ^2}} \end{aligned}$$

assuming that *F* and *B* are such that the normalization condition (25) is satisfied up to ${\mathscr{O}}(\phi ^4)$.

## Scattering problem

Let us assume that a monochromatic *y*-wave is injected into the system through the left PhC arm. The scattering problem can now be solved through the following decomposition of the electric field within the ADL

27 $$\begin{aligned} {\varvec{E}}={\varvec{E}}_{\scriptscriptstyle dir}+{\varvec{E}}_{\scriptscriptstyle res}, \end{aligned}$$

where the direct contribution is simply the electric field of the incident wave

28 $$\begin{aligned} {\varvec{E}}_{\scriptscriptstyle dir}=\sqrt{I_0}e^{ik_oz} \left\{ 0, \ 1\right\} ^{\dagger } \end{aligned}$$

with intensity $I_0$, while ${\varvec{E}}_{\scriptscriptstyle res}$ can be viewed as the contribution due to the resonant pathway via exitation of the resonant eigenmode ${\varvec{E}}_r$. Substituting Eq. (27) into Eq. (1) one obtains the inhomogeneous equation

29 $$\begin{aligned} \partial _z^2{\varvec{E}}_{\scriptscriptstyle res} +k^2_0{\hat{\epsilon }} {\varvec{E}}_{\scriptscriptstyle res}={\varvec{J}} \end{aligned}$$

with

30 $$\begin{aligned} {\varvec{J}}=-\partial _z^2{\varvec{E}}_{\scriptscriptstyle dir} -k^2_0{\hat{\epsilon }} {\varvec{E}}_{\scriptscriptstyle dir}. \end{aligned}$$

Equation (29) can be solved with the use of Green’s function

31 $$\begin{aligned} {\varvec{E}}_{\scriptscriptstyle res}(z)=\int \limits _{-d}^{d}{\widehat{G}}(z,z'){\varvec{J}}(z')dz' \end{aligned}$$

that is defined as the solution of Maxwell’s equations with a delta source

32 $$\begin{aligned} \partial _z^2{\widehat{G}}(z,z') +k^2_0{\hat{\epsilon }} {\widehat{G}}(z,z')=\delta (z-z')\widehat{{\mathbb {I}}}_{2}, \end{aligned}$$

where $\widehat{{\mathbb {I}}}_{2}$ is the $2\times 2$ identity matrix. According to^50^ Green’s function can expanded into the orthonormal resonant eigenmodes as

33 $$\begin{aligned} {\widehat{G}}(z,z')=\sum _n\frac{{\varvec{E}}_n(z) \otimes {\varvec{E}}_n(z')}{2k_0(k_0-k_n)}. \end{aligned}$$

Of course we do not know the full spectrum $k_n$, since Eqs. (16, 17) are not solved analytically. We, however, assume that the contribution of all eigenfields except ${\varvec{E}}_{\scriptscriptstyle res}$ is accumulated in the direct field. Thus, in the spectral vicinity of the quasi-BIC we apply the *resonant approximation* taking into account only the eigenmode associated with the BIC

34 $$\begin{aligned} {\widehat{G}}_{\scriptscriptstyle res}(z,z')=\frac{{\varvec{E}}_{r}(z) \otimes {\varvec{E}}_{r}(z')}{2k_0(k_0-k_{r})}. \end{aligned}$$

The resonant field can now be calculated from Eq. (31) with the resonant Green’s function Eq. (34) once the FV normalization condition (26) is applied to the quasi-BIC eigenmode. The analytic expression for the resonant field reads

35 $$\begin{aligned} \varvec{E}_{\scriptscriptstyle res}(z)= & {} \frac{1}{k_0(k_0-k_r) (F+B\cdot \phi ^2) \epsilon _e} \left[ \frac{i k_o \sqrt{I_0} k_0^2 (\epsilon _e-\epsilon _o) \cos {(k_o d)}}{k_{o}^2-\pi ^2/4d^2} \cdot \phi + \mathcal{O}(\phi ^3) \right] \nonumber \\&\quad \left\{ \begin{array} {c} \sin {\left( \frac{\pi z}{2 d}\right) }\\ \left[ \sin {\left( \frac{\pi z}{2 d}\right) } + ie^{\frac{i\pi q}{2}} \sin {\left( \frac{\pi q z}{2 d}\right) } \right] \cdot \phi \end{array}\right\} . \end{aligned}$$

**Figure 2:**
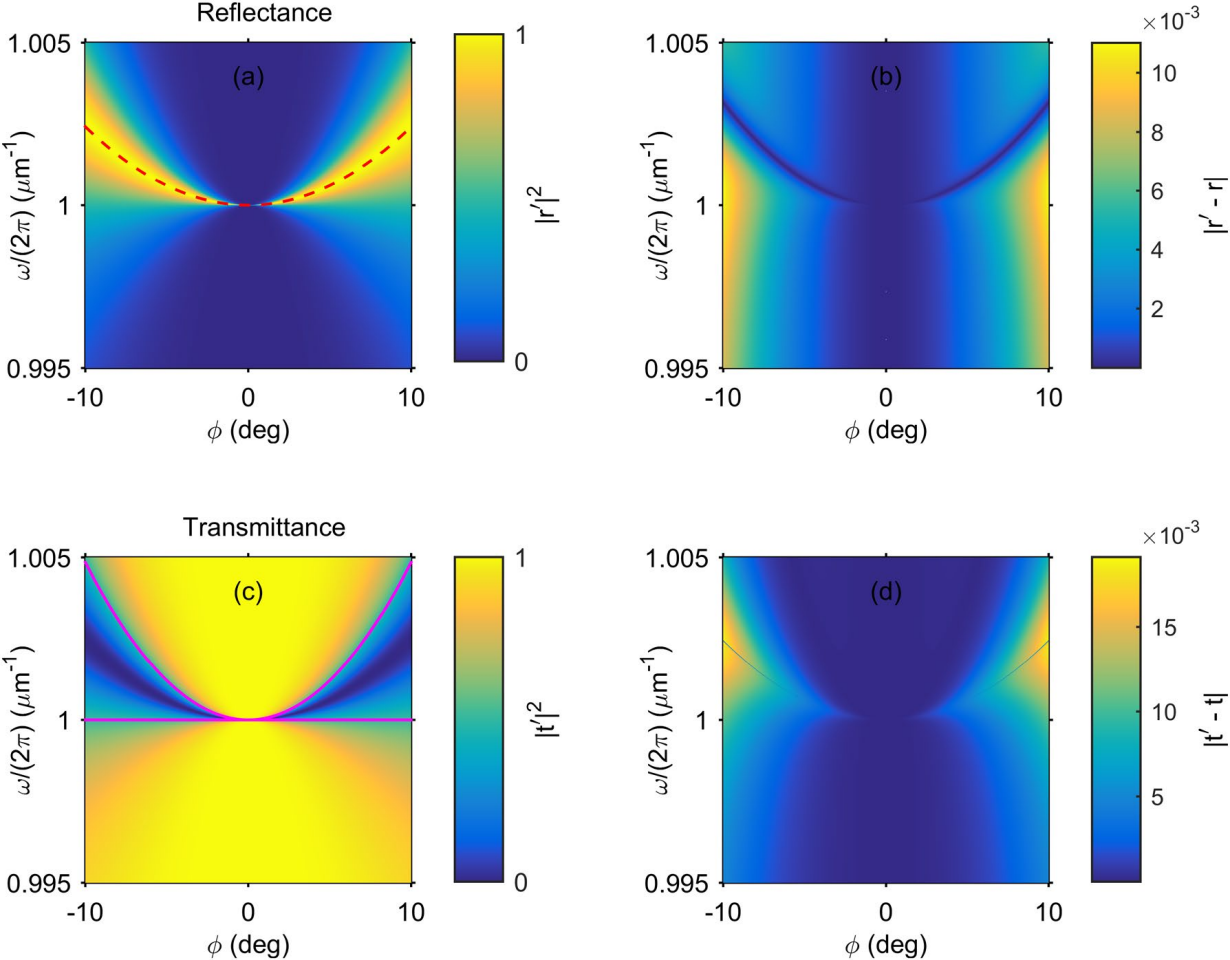
(**a**, **c**) The reflectance $|r^{\prime }|^2$ (**a**) and transmittance $|t^{\prime }|^2$ (**c**) spectra versus tilt angle $\phi$ of *y*-waves for PhC structure from Fig. 1a calculated with Berreman’s method. The dashed red line shows the analytic resonant frequency $\omega _r/(2\pi )$ (22). The solid magenta lines show the analytic results for half-minima in transmittance $(\omega _r \pm \gamma )/(2\pi )$. (**b**, **d**) The difference between reflectance (**b**) and transmittance (**d**) spectra calculated with Berreman’s method and analytically (38), (39). The parameters are the same as in Fig. 1.

The above equation constitutes the perturbative solution of the scattering problem with the accuracy up to $\mathcal{O} (\phi ^3)$. Notice that the terms dependant on *B* also vanish as $\mathcal{O} (\phi ^3)$. Thus, in evaluating the FV normalization condition (25) we can restrict ourselves to $\phi =0$ in which case the eigenmode is a BIC. Further on we safely set $B=0$ in all calculations. The BIC is localized function decaying with $z\rightarrow \pm \infty$. Since the division into the scattering domain and the waveguides is arbitrary and the flux term is vanishing with $z\rightarrow \pm \infty$, one can rewrite the normalization condition for the BIC as follows

36 $$\begin{aligned} \int \limits _{-\infty }^{\infty }{\varvec{E}}_n\cdot {\hat{\epsilon }}{\varvec{E}}_{n}dz=1. \end{aligned}$$

We mention in passing that the equivalence between Eqs. (25) and (36) can also be proven by subsequently applying the Newton–Leibniz axiom and Maxwell’s equations (1) to the flux term in Eq. (25). The integral in Eq. (36) is nothing but the energy stored in the eigenmode up to a constant prefactor. This integral for the system in Fig. 1a has been evaluated analytically in our previous work^45^. As a result the normalization condition (36) yields

37 $$\begin{aligned} F=\frac{d}{1 - q}. \end{aligned}$$

Finally, the reflection and transmission coefficients can be found through the following equations, correspondingly

38 $$\begin{aligned} r=e^{-ik_od}{\varvec{e}}_y^{\dagger } \cdot \left[ {\varvec{E}}(-d)-\sqrt{I_0}e^{-ik_od}{\varvec{e}}_y\right] , \end{aligned}$$

and

39 $$\begin{aligned} t=e^{-ik_od}{\varvec{e}}_y^{\dagger } \cdot {\varvec{E}}(d), \end{aligned}$$

where ${\varvec{e}}_y= \left\{ 0, 1 \right\} ^{\dagger }$ and ${\varvec{E}}$ is computed through Eqs. (27, 28, 35). In Fig. 2 we compare the perturbative analytic solution against direct numerical simulations with the Berreman transfer-matrix method^54^. One can see that deviation is no more than 2% even for relatively large angle $\phi = 10$ deg. In Fig. 2 one can see a typical Fano transmission pattern with interference between two pathways^3^. The direct pathway is due to the incident ordinary wave penetrating through the ADL from the left to the right PhC arm. The second pathway is the resonant excitation of the quasi-BIC. Finally, the origin of the collapse of the Fano resonance^35–40^ at the exact normal incidence is easily explained by the denominator $k_0-k_r$ in Eq. (35) which contains a vanishing resonant width $\gamma$.

## Conclusion

In this paper we considered light scattering by an anisotropic defect layer embedded into anisotropic photonic crystal in the spectral vicinity of an optical BIC. Using a resonant state expansion method we derived an analytic solution for reflection and transmission amplitudes. The analytic solution is constructed via a perturbative approach with the BIC as the zeroth order approximation. The solution is found to accurately describe the collapsing Fano feature in the spectral vicinity of the BIC. So far the theoretical attempts to describe the Fano feature induced by the BIC relied on phenomenological approaches such as the *S*-matrix approach^38^, or the coupled mode theory^39^. To the best of our knowledge this is the first full-wave analytic solution involving an optical BIC reported in the literature. We believe that the results presented offer a new angle onto the resonant state expansion method paving a way to analytic treatment of resonant scattering due to optical BICs. In particular we expect that the resonant approximation can be invoked to build a rigorous theory of nonlinear response^55^. Recently, the Fano resonance induced by an optical BIC in a liquid crystal ADL has been reported experimentally in^56^ at the Brewster angle. We believe that extending our theoretical approach to the Brewster BIC could be an interesting topic for the future studies. Nowadays, the BICs in photonic systems have already found important applications in enhanced optical absorbtion^57^, surface enhanced Raman spectroscopy^58^, lasing^59^, and sensors^60^. We speculate that analytic results are of importance for a further insight into localization of light as well as the concurrent phenomenon of collapsing Fano resonance.

## Data Availability

The data that support the findings of this study are available from the corresponding author, P.S.P., upon reasonable request.

## References

[1] Joannopoulos JD, Johnson SG, Winn JN, Meade RD (2011). Photonic Crystals: Molding the Flow of Light.

[2] Yang Y (2015). Nonlinear Fano-resonant dielectric metasurfaces. Nano Letters.

[3] Miroshnichenko AE, Flach S, Kivshar YS (2010). Fano resonances in nanoscale structures. Rev. Mod. Phys..

[4] Lalanne P, Yan W, Vynck K, Sauvan C, Hugonin J-P (2018). Light interaction with photonic and plasmonic resonances. Laser Photonics Rev..

[5] Stratton JA (1941). Electromagnetic Theory.

[6] Haus HA (1984). Waves and Fields in Optoelectronics.

[7] Fan S, Suh W, Joannopoulos JD (2003). Temporal coupled-mode theory for the Fano resonance in optical resonators. J. Opt. Soc. Am. A.

[8] Suh W, Wang Z, Fan S (2004). Temporal coupled-mode theory and the presence of non-orthogonal modes in lossless multimode cavities. IEEE J. Quantum Electron..

[9] Ruan Z, Fan S (2009). Temporal coupled-mode theory for Fano resonance in light scattering by a single obstacle. J. Phys. Chem. C.

[10] Ruan Z, Fan S (2012). Temporal coupled-mode theory for light scattering by an arbitrarily shaped object supporting a single resonance. Phys. Rev. A.

[11] Rotter I (1991). A continuum shell model for the open quantum mechanical nuclear system. Rep. Prog. Phys..

[12] Dittes F (2000). The decay of quantum systems with a small number of open channels. Phys. Rep..

[13] Ołowicz J, Płoszajczak M, Rotter I (2003). Dynamics of quantum systems embedded in a continuum. Phys. Rep..

[14] Chruściński D, Kossakowski A (2013). Feshbach projection formalism for open quantum systems. Phys. Rev. Lett..

[15] Stöckmann H-J (1999). Quantum Chaos: An Introduction.

[16] Pichugin K, Schanz H, Šeba P (2001). Effective coupling for open billiards. Phys. Rev. E.

[17] Stöckmann H-J (2002). Effective Hamiltonian for a microwave billiard with attached waveguide. Phys. Rev. E.

[18] Sadreev AF, Rotter I (2003). S-matrix theory for transmission through billiards in tight-binding approach. J. Phys. A Math. Gen..

[19] Savin D, Sokolov V, Sommers H-J (2003). Is the concept of the non-Hermitian effective Hamiltonian relevant in the case of potential scattering?. Phys. Rev. E.

[20] Maksimov DN, Sadreev AF, Lyapina AA, Pilipchuk AS (2015). Coupled mode theory for acoustic resonators. Wave Motion.

[21] Racec PN, Racec ER, Neidhardt H (2009). Evanescent channels and scattering in cylindrical nanowire heterostructures. Phys. Rev. B.

[22] Gongora JST, Favraud G, Fratalocchi A (2017). Fundamental and high-order anapoles in all-dielectric metamaterials via Fano-Feshbach modes competition. Nanotechnology.

[23] Lee H, Reichl LE (2010). R-matrix theory with Dirichlet boundary conditions for integrable electron waveguides. J. Phys. A Math. Theor..

[24] Schanz H (2003). Reaction matrix for Dirichlet billiards with attached waveguides. Physica E Low Dimens. Syst. Nanostruct..

[25] Berenger J-P (1994). A perfectly matched layer for the absorption of electromagnetic waves. J. Comput. Phys..

[26] Chew WC, Weedon WH (1994). A 3D perfectly matched medium from modified Maxwell's equations with stretched coordinates. Microw. Opt. Technol. Lett..

[27] Civitarese O, Gadella M (2004). Physical and mathematical aspects of Gamow states. Phys. Rep..

[28] More RM (1971). Theory of decaying states. Phys. Rev. A.

[29] Snyder AW, Love J (2012). Optical Waveguide Theory.

[30] Muljarov EA, Langbein W, Zimmermann R (2010). Brillouin-Wigner perturbation theory in open electromagnetic systems. EPL (Europhys. Lett.).

[31] Kristensen PT, Hughes S (2013). Modes and mode volumes of leaky optical cavities and plasmonic nanoresonators. ACS Photonics.

[32] Sauvan C, Hugonin JP, Maksymov IS, Lalanne P (2013). Theory of the spontaneous optical emission of nanosize photonic and plasmon resonators. Phys. Rev. Lett..

[33] Hsu CW, Zhen B, Stone AD, Joannopoulos JD, Soljačić M (2016). Bound states in the continuum. Nat. Rev. Mater..

[34] Koshelev K, Favraud G, Bogdanov A, Kivshar Y, Fratalocchi A (2019). Nonradiating photonics with resonant dielectric nanostructures. Nanophotonics.

[35] Kim CS, Satanin AM, Joe YS, Cosby RM (1999). Resonant tunneling in a quantum waveguide: effect of a finite-size attractive impurity. Phys. Rev. B.

[36] Shipman SP, Venakides S (2005). Resonant transmission near nonrobust periodic slab modes. Phys. Rev. E.

[37] Sadreev AF, Bulgakov EN, Rotter I (2006). Bound states in the continuum in open quantum billiards with a variable shape. Phys. Rev. B.

[38] Blanchard C, Hugonin J-P, Sauvan C (2016). Fano resonances in photonic crystal slabs near optical bound states in the continuum. Phys. Rev. B.

[39] Bulgakov EN, Maksimov DN (2018). Optical response induced by bound states in the continuum in arrays of dielectric spheres. J. Opt. Soc. Am. B.

[40] Bogdanov AA (2019). Bound states in the continuum and Fano resonances in the strong mode coupling regime. Adv. Photonics.

[41] Foley JM, Young SM, Phillips JD (2014). Symmetry-protected mode coupling near normal incidence for narrow-band transmission filtering in a dielectric grating. Phys. Rev. B.

[42] Cui X, Tian H, Du Y, Shi G, Zhou Z (2016). Normal incidence filters using symmetry-protected modes in dielectric subwavelength gratings. Sci. Rep..

[43] Doskolovich LL, Bezus EA, Bykov DA (2019). Integrated flat-top reflection filters operating near bound states in the continuum. Photonics Res..

[44] Nguyen TG, Yego K, Ren G, Boes A, Mitchell A (2019). Microwave engineering filter synthesis technique for coupled ridge resonator filters. Opt. Express.

[45] Timofeev IV, Maksimov DN, Sadreev AF (2018). Optical defect mode with tunable $$Q$$-factor in a one-dimensional anisotropic photonic crystal. Phys. Rev. B.

[46] Rytov SM (1956). Electromagnetic properties of a finely stratified medium. Sov. Phys. JETP.

[47] Yariv A, Yeh P (1984). Optical Waves in Crystals.

[48] Shi H, Tsai C-H (1984). Polariton modes in superlattice media. Solid State Commun..

[49] Camley RE, Mills DL (1984). Collective excitations of semi-infinite superlattice structures: surface plasmons, bulk plasmons, and the electron-energy-loss spectrum. Phys. Rev. B.

[50] Doost MB, Langbein W, Muljarov EA (2013). Resonant state expansion applied to two-dimensional open optical systems. Phys. Rev. A.

[51] Doost MB, Langbein W, Muljarov EA (2014). Resonant-state expansion applied to three-dimensional open optical systems. Phys. Rev. A.

[52] Muljarov EA, Weiss T (2018). Resonant-state expansion for open optical systems: generalization to magnetic, chiral, and bi-anisotropic materials. Opt. Lett..

[53] Tanimu A, Muljarov EA (2018). Resonant-state expansion applied to one-dimensional quantum systems. Phys. Rev. A.

[54] Berreman DW (1972). Optics in stratified and anisotropic media: $$4 \times 4$$-matrix formulation. J. Opt. Soc. Am..

[55] Bulgakov EN, Maksimov DN (2019). Nonlinear response from optical bound states in the continuum. Sci. Rep..

[56] Pankin P (2020). One-dimensional photonic bound states in the continuum. Commun. Phys..

[57] Zhang J (2015). Plasmonic focusing lens based on single-turn nano-pinholes array. Opt. Express.

[58] Romano S (2018). Surface-enhanced Raman and fluorescence spectroscopy with an all-dielectric metasurface. J. Phys. Chem. C.

[59] Kodigala A (2017). Lasing action from photonic bound states in continuum. Nature.

[60] Romano S (2018). Label-free sensing of ultralow-weight molecules with all-dielectric metasurfaces supporting bound states in the continuum. Photonics Res..

